# Light-Regulated Cancer Immunotherapy Using Individually Encapsulated Synthetic Circuit–Engineered Cells

**DOI:** 10.21769/BioProtoc.5770

**Published:** 2026-07-20

**Authors:** Yirui Han, Han Lu, Yue Zhao, Kun Fu, Guangjun Nie, Yazhou Chen

**Affiliations:** 1 Henan Institute of Advanced Technology, Zhengzhou University, Zhengzhou, China; 2 Department of Oral and maxillofacial surgery, First Affiliated Hospital of Zhengzhou University, Zhengzhou, China; 3 CAS Key Laboratory for Biomedical Effects of Nanomaterials & Nanosafety, CAS Center for Excellence in Nanoscience, National Center for Nanoscience and Technology, Beijing, China

**Keywords:** Single-cell encapsulation, Optogenetic switch, Cell therapy, Cell surface engineering, Enzyme-mediated crosslinking, Cytocompatible coating, Synthetic circuit–engineered cells

## Abstract

Cell therapy holds great promise for cancer immunotherapy, but its clinical efficacy is severely hindered by poor post-transplant cell survival, low homing efficiency, and host immune clearance. To address these challenges, this study develops a novel light-controlled immunotherapy strategy that integrates a red/far-red light genetic switch with single-cell encapsulation engineering. The red/far-red light (660/730 nm) reversible regulatory system enables precise spatiotemporal control over the expression of therapeutic proteins in engineered cells (e.g., CAR-T or engineered HEK 293T cells), allowing on-demand activation of anti-tumor immune responses. On this basis, a mild enzyme-mediated single-cell encapsulation technique is further employed to rapidly form a protective hydrogel coating in situ on the cell surface, thereby enhancing the survival of transplanted cells under hostile in vivo microenvironments. This strategy combines precise gene expression regulation with physical protection, improving therapeutic outcomes without the need for genomic modification of the cells. It provides a new paradigm for developing safe, controllable, and efficient cancer immunotherapy.

Key features

• Using a 660/730 nm red/far-red light reversible switch, deep tissue penetration enables spatiotemporal precise control of tumor-targeted therapeutic proteins.

• Achieving rapid and gentle in situ gelation encapsulation of single-cell surfaces through HRP-pHLIP membrane anchoring and HA-dopamine enzymatic crosslinking.

• Targeted strategies to overcome post-transplant hypoxia, inflammatory stress, and pulmonary first-pass entrapment, physically enhancing early cell survival prior to reaching the target tissue.

• This experimental protocol requires at least three days.

## Graphical overview


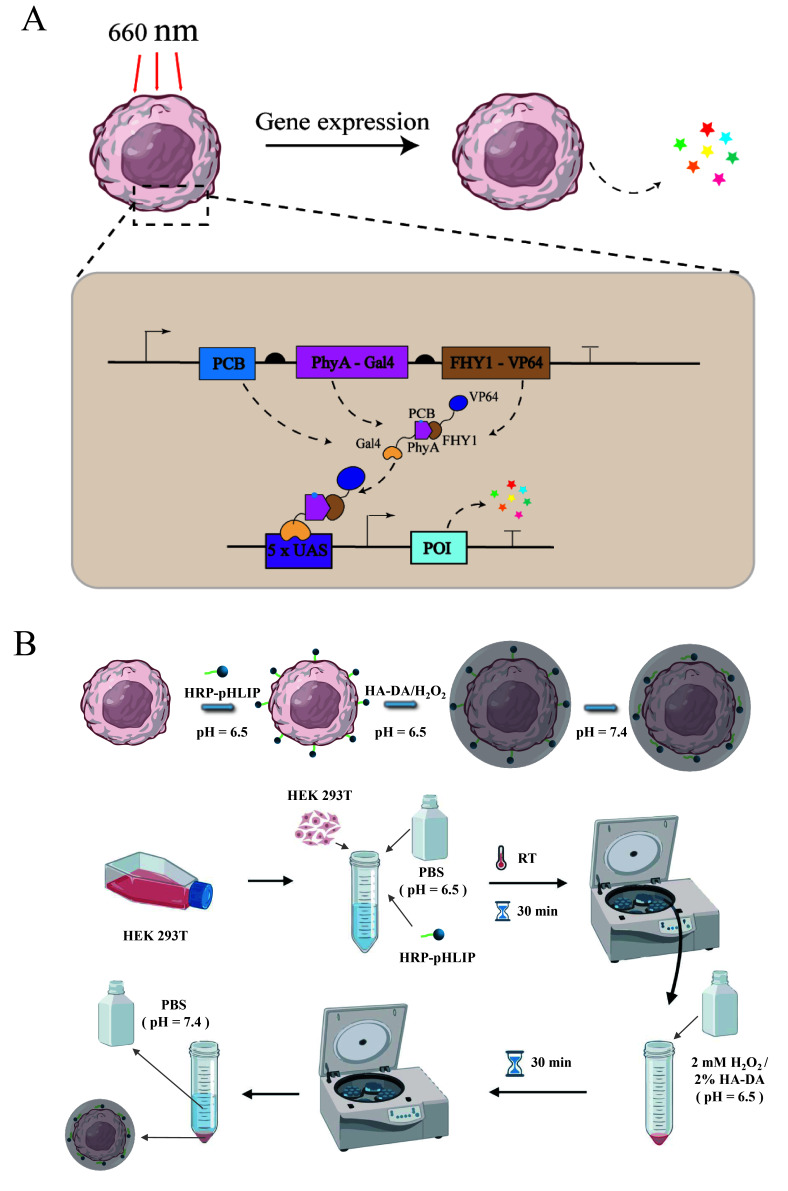


**Schematic illustration of encapsulating synthetic circuit–engineered cells.** (A) Schematic representation of the optogenetic control system, illustrating the light-induced (660 nm) association and (730 nm) dissociation of PhyA and FHY1. The opto-genetically controlled gene regulation system employs two modular components: (1) a light-dependent transactivator (FHY1-VP64) created by fusing the PhyA interaction domain FHY1 with a tetrameric VP16 activation domain (VP64), driven by a constitutive promoter, and (2) a fusion light sensor (ΔPhyA-Gal4) formed by conjugating phytochrome PhyA with the Gal4 DNA-binding domain. Under 660 nm illumination in the presence of PCB chromophore, FHY1-VP64 binds ΔPhyA-Gal4, enabling the complex to activate transgene expression via the synthetic P5×UAS-PhCMVmin promoter. Far-red light (730 nm) triggers complex dissociation, terminating expression. (B) Schematic illustration of cell surface encapsulation engineering. HEK293T cells are collected and resuspended in phosphate-buffered saline (PBS, pH = 6.5) containing HRP-pHLIP. Then, the mixture is incubated at room temperature for 30 min. Subsequently, the mixture is centrifuged at 150× *g* for 5 min and washed three times with PBS to remove any unbound HRP-pHLIP. For encapsulation, labeled cells are mixed with a solution containing 2 mM of H_2_O_2_ and 2% (mass/volume) hyaluronic acid–dopamine (HA-DA) hydrogel solution and gently stirred for 30 min. After the stirring, cells are resuspended and washed three times with PBS (pH = 7.4) to remove any residual reagents.

## Background

Cell therapy has been widely explored in regenerative medicine and cancer immunotherapy [1]. By administering living cells with defined therapeutic functions, such as stem cells or immune cells, this approach aims to repair damaged tissues, modulate immune responses, or eliminate malignant cells [2–5]. Among these modalities, chimeric antigen receptor T-cell (CAR-T) therapy has achieved remarkable clinical success in hematologic malignancies and has provided an effective option for patients with relapsed or refractory disease [6–7]. Likewise, mesenchymal stem cells (MSCs), owing to their immunomodulatory, tissue-reparative, and homing properties, have shown promise in clinical studies of autoimmune diseases, myocardial infarction, and graft-versus-host disease (GVHD), with more than one thousand registered trials worldwide [8–10].

Despite these advances, poor post-transplant survival and limited homing efficiency remain major barriers to clinical translation [11]. Following intravenous infusion or local delivery, transplanted cells are exposed to hypoxia, ischemia, and inflammatory stress, resulting in substantial cell loss before reaching the target tissue. For example, more than 90% of intravenously administered MSCs are trapped in the lungs within 24 h, and only a small fraction successfully home to target organs [12]. For CAR-T cells, the physical barriers of solid tumors, aberrant vasculature, and immunosuppressive tumor microenvironments likewise restrict infiltration and persistence [13–14]. In addition, allogeneic cell products, including off-the-shelf CAR-T cells and donor-derived MSCs, may be rapidly cleared by host immune responses, thereby limiting therapeutic efficacy [15].

To overcome these limitations, researchers have been actively exploring novel strategies for spatiotemporally precise regulation of therapeutic cell functions [16]. In recent years, optogenetic-based gene switch technologies, particularly the red/far-red light reversible regulatory system, have provided a new solution for cancer immunotherapy. Compared with blue light systems, red light exhibits deeper tissue penetration (up to several centimeters) and lower photocytotoxicity, making it more suitable for in vivo applications. A typical red light–controlled gene switch utilizes phytochromes (e.g., *Arabidopsis* PhyB) and their interacting factors (PIFs). Under red light irradiation (660 nm), phytochrome binds to PIF, thereby initiating downstream gene transcription; upon far-red light irradiation (730 nm), they dissociate, leading to rapid termination of gene expression [17]. This red/far-red light bidirectional switch features high spatiotemporal resolution, reversibility, and fast response kinetics. It has been successfully applied to regulate the expression of therapeutic cytokines (e.g., IL-2, IFN-γ) in CAR-T cells, the on-demand release of immune checkpoint inhibitors, and the membrane localization of chimeric antigen receptors. Compared with conventional constitutive expression or chemically inducible systems, the red light–controlled gene switch enables the stringent restriction of therapeutic protein expression to the tumor site and within a defined time window, thereby substantially reducing systemic immune-related toxicities. When integrated into the synthetic gene circuits of engineered cells, such optogenetic switches allow remote and reversible control over immune response intensity, opening a new avenue for precision cancer immunotherapy.

Meanwhile, cell surface engineering has also attracted much attention as an important strategy for improving the survival and function of transplanted cells [18–19]. As the interface between cells and their environment, the cell surface plays a central role in adhesion, signaling, immune recognition, and homing. Surface engineering enables the introduction of additional functions without altering the cell genome, thereby allowing precise control over cellular behavior [20]. Existing approaches include physical encapsulation with biocompatible coatings, chemical conjugation of functional molecules, and metabolic incorporation of non-native groups into membrane components [21–23]. Protective coatings based on hydrogels or polyelectrolyte membranes, as well as membrane-anchored immunomodulatory molecules, can reduce immune recognition and prolong the in vivo persistence of allogeneic cells. Such coatings may also act as local delivery platforms, creating a supportive pericellular microenvironment that enhances cell survival and functional stability [24–25].

In this protocol, we developed a cell surface encapsulation strategy based on the HRP-pH-low insertion peptide (pHLIP). HRP-pHLIP was inserted into the cell membrane, and in the presence of H_2_O_2_, horse radish peroxidase (HRP) catalyzed the crosslinking of hyaluronic acid–dopamine (HA-DA), enabling in situ encapsulation of individual cells. This approach integrates targeted membrane insertion with enzyme-mediated crosslinking to generate a mild, controllable protective coating, with the goal of improving cell survival under hostile post-transplantation conditions.

## Materials and reagents

**Biological materials**

1. *E. coli* BL21 (DE3)

2. HEK 293T (human embryonic kidney 293T cells) (Servicebio STCC10301P-1)

**Reagents**

1. Hyaluronic acid (HA) (Sigma, CAS: 9067-32-7)

2. Dopamine (DA) (Sigma, CAS: 62-31-7)

3. 1-ethyl-3-(3-dimethylaminopropyl) carbodiimide (EDC) (Sigma, CAS: 1892-57-5)

4. N-hydroxysuccinimide (NHS) (Sigma, CAS: 25389-94-0)

5. PBS (Gibco, CAS: 10010023)

6. Deuterium oxide (D_2_O) (Sigma, CAS: 7789-20-0)

7. Kanamycin (Sigma, CAS: 69-52-3)

8. MultiS One Step Cloning Kit (Vazyme, CAS: C113-01)

9. Imidazole (Sigma, CAS: 288-32-4)

10. NaCl (Sigma, CAS: 7647-14-5)

11. NaH_2_PO_4_·2H_2_O (Sigma, CAS: 7558-79-4)

12. Agar (Sigma, CAS: 9002-18-0)

13. H_2_O_2 _(Sigma, CAS: 7722-84-1)

14. High-glucose DMEM (Gibco, catalog number: C11995500BT)

15. FBS (Gibco, catalog number: A5256701)

16. Penicillin/streptomycin (Pricella Inc., catalog number: PB180120)

17. Lipo293^TM ^(Beyotime, catalog number: C0521)

18. Yeast (Sigma, CAS: 8013-01-2)

19. Tryptone (Sigma, CAS: 91079-40-2)

20. Ni-NTA (Smart-Lifesciences, CAS: SA005025)

21. Hydrochloric acid (Sigma, CAS: 7647-01-0)

22. Sodium hydroxide (Sigma, CAS:1310-73-2)

**Solutions**

1. Luria-Bertani (LB) medium (see Recipes)

2. Solid LB medium (see Recipes)

3. Isopropyl β-D-thiogalactopyranoside (IPTG) (see Recipes)

4. Lysis buffer (see Recipes)

5. Wash buffer (see Recipes)

6. Elution buffer (see Recipes)

**Recipes**

**1. LB medium**

**Table:** 

Reagent	Final concentration	Quantity or volume
Tryptone	1 g/100 mL	1 g
Yeast extract	0.5 g/100 mL	0.5 g
NaCl	1 g/100 mL	1 g
H_2_O	n/a	100 mL

Prepare the LB medium by mixing 1 g of tryptone, 0.5 g of yeast extract, and 1 g of NaCl. Then, sterilize it under high pressure at 121 °C for 60 min.

**2. Solid LB medium**

**Table:** 

Reagent	Final concentration	Quantity or volume
Agar	1.5 g/100 mL	1.5 g
LB medium	n/a	100 mL

Add 1.5 g/100 mL of agar powder (agar) to the LB medium. Sterilize at 121 °C under high pressure for 60 min. When the temperature cools down to 50 °C, pour it into a sterile Petri dish and let it cool down for later use.

**3. IPTG**

**Table:** 

Reagent	Final concentration	Quantity or volume
IPTG	1 M	2.3 g
Double-distilled water (ddH_2_O)	n/a	10 mL

Weigh 2.3 g of IPTG, add it to 10 mL of ddH_2_O, and filter-sterilize the resulting solution through a 0.22 μm membrane filter.

**4. Lysis buffer**

**Table:** 

Reagent	Final concentration	Quantity or volume
NaH_2_PO_4_·2H_2_O	50 mM	7.8 g
NaCl	300 mM	17.54g
Imidazole	10 mM	0.68 g
ddH_2_O	n/a	1 L

Filter-sterilize.

**5. Wash buffer**

**Table:** 

Reagent	Final concentration	Quantity or volume
NaH_2_PO_4_·2H_2_O	50 mM	7.8 g
NaCl	300 mM	17.54 g
Imidazole	20 mM	1.36 g
ddH_2_O	n/a	1 L

Filter-sterilize.

**6. Elution buffer**

**Table:** 

Reagent	Final concentration	Quantity or volume
NaH_2_PO_4_·2H_2_O	50 mM	7.8 g
NaCl	300 mM	17.54 g
Imidazole	250 mM	17.0 g
ddH_2_O	n/a	1 L

Filter-sterilize.

## Equipment

1. Refrigerated constant temperature air bath shaker (Jintan Xuri Experimental Instrument Factory, Hangzhou, China, model: JTYS-2000-L)

2. Ice maker (Xueke Electric Appliance Co., Ltd., Changshu, China, model: IMS-50)

3. Water purifier (Youpu Ultra-pure Technology Co., Ltd., Sichuan, China, model: UPH-I-30T)

4. Electrothermal constant temperature water bath (Yiheng Scientific Instrument Co., Ltd., Shanghai, China, model: HWS-12)

5. Ultrasonic homogenizer (Lichen Instrument Technology Co., Ltd., Shanghai, China, model: LJY96-IIN)

6. Centrifuge (Eppendorf, model: 5804R)

7. Point-scanning confocal (e.g., Zeiss, model: LSM800 or Leica, model: SP8)

8. Freeze-drying machine (Yaxing Instrument, model: LGJ-10N/B)

9. DC Power Supply (Zhaoxin, model: RXN-305D)

## Procedure

**A. Synthesis and characterization of HA-DA**

**Figure 1.**
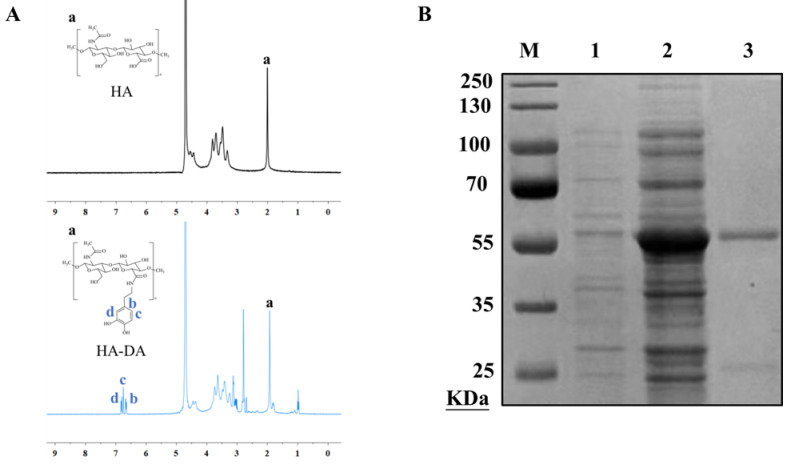
Proton nuclear magnetic resonance (^1^H NMR) spectra of hyaluronic acid–dopamine (HA-DA) conjugates and purification of HRP-pHLIP [26]. (A) ^1^H NMR spectra of HA-DA conjugates. a) characteristic peaks of hyaluronic acid; b,c,d) characteristic peaks of dopamine (B) Lane M: protein molecular weight marker. Lane 1: *E. coli* BL21 (DE3) cells without induction. Lane 2: *E. coli* BL21 (DE3) cells after induction. Lane 3: Purified HRP-pHLIP.

1. Weigh 1 g of HA (molecular weight 12 kDa) and dissolve in 100 mL of PBS. Gently stir the solution at room temperature until complete dissolution.

2. To activate the carboxyl groups on the HA molecular chains, add EDC to a final concentration of 5 mM, followed by NHS to a final concentration of 10 mM.

3. Adjust the pH of the reaction mixture to 5.0 using diluted hydrochloric acid (10 mM) or sodium hydroxide solution (10 mM) and allow the reaction to proceed under continuous stirring at room temperature for 6 h.

4. Add DA to a final concentration of 10 mM, and stir the reaction continuously at room temperature for 24 h under a nitrogen atmosphere to prevent oxidation of dopamine during the reaction (in a vacuum valve reaction flask).

5. After the reaction is completed, transfer the crude product to a dialysis bag with a molecular weight cutoff of 14 kDa and dialyze against deionized water at 4 °C for 72 h.

6. Change the dialysis medium every 8 h to thoroughly remove unreacted small molecules and by-products.

7. Following dialysis, freeze-dry the purified solution to obtain the final product, the HA-DA conjugate, as a spongy solid.

8. Confirm the chemical structure of the synthesized product using proton nuclear magnetic resonance (^1^H NMR) spectroscopy. The HA-DA conjugate is dissolved in D_2_O.

9. Determine successful grafting of dopamine and grafting efficiency based on the chemical shifts and integrate the peak areas of characteristic signals (Figure 1A).

**B. Gene cloning, protein expression, and purification of HRP-pHLIP**

1. Target genes encoding horseradish peroxidase (HRP, NCBI accession number: HW399451.1) and the HRP-pHLIP fusion protein were custom-synthesized by Genewiz Biotechnology Co., Ltd. (Suzhou, China) with the pHLIP domain containing the optimized nucleotide sequence: 5’-GCGGAGCAGAACCCGATTTATTGGGCGCGCTATGCGGATTGGCTGTTTACCACCCCGCTGTTACTGCTGGATCTGGCGCTGCTGGTGGATGCGGATGAAGGCACC-3’.

2. Subclone the synthesized products into the pET28a (+) expression vector using conventional restriction enzyme cloning to obtain the recombinant plasmid pET28a-HRP-pHLIP. The correctly sequenced recombinant plasmid is used for subsequent transformation experiments.

3. Transform the sequence-verified pET28a-HRP-pHLIP plasmid into *Escherichia coli* BL21 (DE3) competent cells using the heat shock method (42 °C for 45 s).

4. Plate the transformed cells onto LB agar plates containing 60 μg/mL kanamycin and incubate overnight at 37 °C. Select a single colony and inoculate into 5 mL of LB liquid medium containing 60 μg/mL kanamycin, followed by incubation at 37 °C with shaking at 200 rpm for 16 h.

5. The following day, transfer the culture at 1% (v/v) inoculum into 30 mL of fresh LB medium (containing 60 μg/mL kanamycin) in a 250 mL conical flask and incubate it under the same conditions until mid-log phase (OD_600_ ≈ 0.8).

6. Then, add IPTG to a final concentration of 0.5 mM to induce protein expression, and incubate the culture for an additional 8 h at 16 °C with shaking at 220 rpm to promote soluble protein expression.

7. Following induction, centrifuge the bacterial culture at 8,000× *g* for 10 min at 4 °C to collect the cell pellet. Resuspend the pellets in pre-chilled lysis buffer.

8. Place the cell suspension in an ice-water bath and subject it to ultrasonication at 30% amplitude using cycles of 5 s on and 5 s off for a total duration of 15 min to fully release soluble intracellular proteins.

9. Then, centrifuge the lysate at 12,000× *g* for 30 min at 4 °C and collect the supernatant.

10. Purify the His-tagged target protein (His-pHLIP-HRP) using Ni-NTA affinity chromatography.

11. After filling the Ni NTA column, add five times the column volume of lysis buffer to the column for equilibration, aiming to make the buffer system of the packing material the same as that of the target protein. Repeat it 2–3 times.

12. After the column is equilibrated, add the obtained soluble protein solution, adjust the flow rate, and keep the sample in the column for at least 2 min to ensure sufficient contact between the sample and the medium. Collect the effluent and repeat this process 2–3 times.

13. Then, perform elution. Add 10 times the column volume of wash buffer to the column to remove nonspecifically adsorbed contaminants from the column. Collect the elution liquid.

14. After elution, add 5–10 times the column volume of elution buffer to the column to wash the target protein. Collect the effluent during the elution process and test it separately.

15. After the target protein elution is completed, first rinse with three times the column volume of lysis buffer, then rinse with five times the column volume of pure water, and finally rinse with two times the column volume of 20% ethanol. Place the rinsed medium at 2–8 °C for storage.

16. Collect the effluent containing the target protein (Figure 1B).

**C. Cell culture and transfection**

1. Culture HEK 293T cell in high-glucose DMEM supplemented with 10% (v/v) heat-inactivated FBS and 1% (v/v) penicillin/streptomycin.

2. For transient transfection, seed HEK 293T cells in 6-well plates at a density of 5 × 10^5^ cells/well and allow them to adhere overnight, achieving 70%–80% confluence prior to transfection.

3. Before carrying out the following transfection steps, replace the culture medium in each well of the 6-well plate containing cells with 1 mL of fresh culture medium (containing serum but no antibiotics).

*Note: Fresh culture medium containing serum and antibiotics can also be used, but the presence of antibiotics may cause certain cytotoxicity in some cells after transfection.*

4. For each cell in the 6-well plate to be transfected, take two clean and sterile centrifuge tubes, add 125 µL of DMEM culture medium without antibiotics and serum to each tube, then add 2.5 μg of plasmid DNA [pYZ484 (carrying the optogenetic induction system, ITR-P_hCMV_-ΔPhyA-Gal4-P2A-FHY1-VP64-pA::P_hCMV_-PcyA-P2A-HO-P2A-Fd-P2A-FNR-pA::P_mpak-_Puro-pA-ITR) and pYH3 (encoding the SEAP expression cassette, ITR-5× UAS-P_hCMVmin_-SEAP-pA::P_mSV40_-BleoR-pA-ITR)] to one of the tubes and gently mix with a pipette; add 4 µL of Lipo293^TM^ transfection reagent to the other tube and gently mix with a pipette.

*Note: Vortexing or centrifugation is not allowed.*

**Figure 2.**
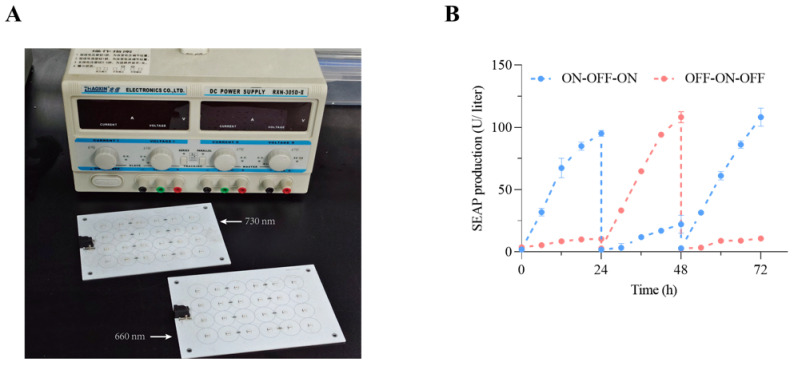
Construction and performance characterization of the optogenetic-control system. (A) Custom LED array (660/730 nm, 1 mW/cm^2^). (B) Analysis of the dynamic characteristics of embryonic alkaline phosphatase (SEAP) secretion by engineered cells. Dynamic release curve of engineered cells after being stimulated by specific wavelength light (660 nm, 5 s; 730 nm, 5 s) within 72 h. Quantify the SEAP concentration every 6 h and replace the complete medium every 24 h.

5. Gently add the culture medium containing DNA to the culture medium containing Lipo293^TM^ transfection reagent, gently invert the centrifuge tube, or gently mix with a pipette. Let it stand at room temperature for 15 min (storing at room temperature for 6 h is stable), and then evenly drop it into the entire well. Gently mix.

6. After approximately 24 h, use a custom LED array (660/730 nm, 1 mW/cm^2^) (Figure 2A).

7. To assess wavelength-dependent regulation, cells received alternating 5 s pulses of 660 nm (“ON” state) and 730 nm (“OFF” state) light at 1 mW/cm^2^ intensity.

8. Following each 24 h stimulation cycle (preceded by medium replacement), cytokine secretion profiles were monitored at 6-h intervals over 72 h (Figure 2B).

**D. Single-cell encapsulation**

**Figure 3.**
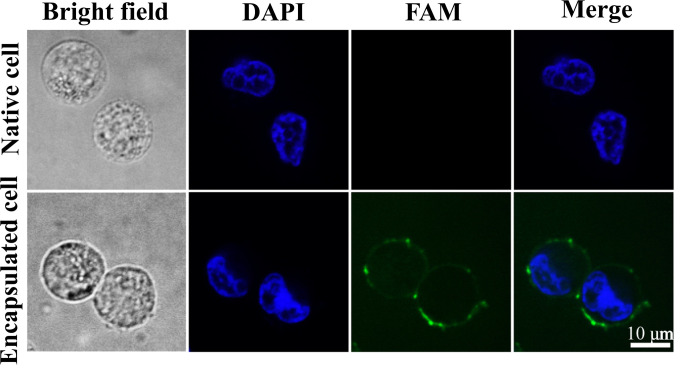
Characterization of encapsulated cells [26] . Fluorescence microscopy images comparing native and encapsulated cells. Green: Carboxyfluorescein (FAM)-labeled hyaluronic acid–dopamine (HA-DA) polymer shell. Blue: DAPI-stained nuclei. Scale bar: 10 μm.

1. Harvest HEK 293T cells (1 × 10^6^ cells) and resuspend them in 1 mL of PBS buffer (pH 6.5) containing HRP-pHLIP fusion protein (50 μg/mL), followed by incubation at room temperature for 30 min.

2. After incubation, centrifuge the cell suspension at 150× *g* for 5 min, discard the supernatant, and wash the cells three times with PBS (pH 6.5) to remove unbound HRP-pHLIP.

3. Resuspend the labeled cells in a hydrogel precursor solution containing 2% (w/v) HA-DA and 2 mM H_2_O_2_ and gently stir it for 30 min to induce HRP-catalyzed crosslinking of HA-DA to form the hydrogel.

4. Collect encapsulated cells by centrifugation at 150× *g* for 5 min, remove the supernatant, and wash the cells three times with PBS (pH 7.4) to remove residual reagents (Figure 3).

## Validation of protocol

This protocol (or parts of it) has been used and validated in the following research article:

- Zhao et al. [26]. On-demand cancer immunotherapy via single-cell encapsulation of synthetic circuit–engineered cells. *Sci Adv*.

## References

[1] Tong Z., Kim C., Ross J. L. and Arvanitis C. (2026). Enhancing immune cell trafficking to brain tumors: Recent advances and therapeutic strategies. Neuro Oncol. e1093/neuonc/noag075. 10.1093/neuonc/noag075 PMC13437905 41934625

[2] Chen J. T., Dadheech N., Tan E. H. P., Ng N. H. J., Koh M. B. C., Shapiro J. and Teo A. K. K. (2025). Stem cell therapies for diabetes. Nat Med. 31(7): 2147 2160 2160. 10.1038/s41591-025-03767-8 40579550

[3] An C., Zhao Y., Guo L., Zhang Z., Yan C., Zhang S., Zhang Y., Shao F., Qi Y., Wang X., Wang H. and Zhang L. (2025). Innovative approaches to boost mesenchymal stem cells efficacy in myocardial infarction therapy. Materials today. Bio. 31: 101476 10.1016/j.mtbio .2025.101476 PMC11787032 39896290

[4] Irvine D. J., Maus M. V., Mooney D. J. and Wong W. W. (2022). The future of engineered immune cell therapies. Science. 378(6622): 853 858 858. 10.1126/science.abq6990 36423279 PMC9919886

[5] Zhang L., Ma X. J. N., Fei Y. Y., Han H. T., Xu J., Cheng L. and Li X. (2022). Stem cell therapy in liver regeneration: Focus on mesenchymal stem cells and induced pluripotent stem cells. Pharmacol. Ther. 232: 108004 10.1016/j.pharmthera .2021.108004 34597754

[6] Tang L., Pan S., Wei X., Xu X. and Wei Q. (2023). Arming CAR-T cells with cytokines and more: Innovations in the fourth-generation CAR-T development. Mol Ther. 31(11): 3146 3162 3162. 10.1016/j.ymthe .2023.09.021 37803832 PMC10638038

[7] Zhao G., Zhao R., Guo K., Li Z., Qin H., Jiang Y., Luo K., Duan P., Liang Y., Zheng X., .(2026). Injectable hydrogel vaccine enhances lymph node immunity for cancer immunotherapy. Nano Today. 10.1016/j.nantod.2026.103048

[8] Liu H., Liu S., Qiu X., Yang X., Bao L., Pu F., Liu X., Li C., Xuan K., Zhou J., .(2020). Donor MSCs release apoptotic bodies to improve myocardial infarction via autophagy regulation in recipient cells. Autophagy. 16(12): 2140 2155 2155. 10.1080/15548627.2020 .1717128 31959090 PMC7751634

[9] Zhang H., Yang K., Chen Y., Jin H. and Liu Q. (2018). The Role of Nestin+ Mesenchymal Stem Cells(MSCs) in Bone Marrow Chronic Graft-Versus-Host Disease. Blood. 132: 2039 2039 2039. 10.1182/blood-2018-99-113230

[10] Lin X., Cai L., Nie M., Wu X., Liang G., Shang L. and Zhao Y. (2023). Light-activated extracellular matrix microcarriers with engineered MSCs loading for autoimmune psoriasis treatment. Chem Eng J. 470: 144118 10.1016/j.cej .2023.144118

[11] Wang Y., Suarez E. R., Kastrunes G., de Campos N. S. P., Abbas R., Pivetta R. S., Murugan N., Chalbatani G. M., V. D’Andrea, Marasco W. A., .(2024). Evolution of cell therapy for renal cell carcinoma. Mol Cancer. 23(1): e1186/s12943–023–01911–x. 10.1186/s12943-023-01911-x PMC10775455 38195534

[12] Wang X., Chen H., Zeng X., Guo W., Jin Y., Wang S., Tian R., Han Y., Guo L., Han J., .(2019). Efficient lung cancer-targeted drug delivery via a nanoparticle/MSC system. Acta Pharmaceutica Sinica. B 9(1): 167 176 176. 10.1016/j.apsb .2018.08.006 30766788 PMC6362298

[13] Jin Y., Huang Y., Ren H., Huang H., Lai C., Wang W., Tong Z., Zhang H., Wu W., Liu C., .(2024). Nano-enhanced immunotherapy: Targeting the immunosuppressive tumor microenvironment. Biomaterials. 305: 122463 10.1016/j.biomaterials .2023.122463 38232643

[14] Guo Y., Hu P. and Shi J. (2024). Nanomedicine Remodels Tumor Microenvironment for Solid Tumor Immunotherapy. J Am Chem Soc. 146(15): 10217 10233 10233. 10.1021/jacs.3c14005 38563421

[15] Grauwet K., Berger T., Kann M. C., Silva H., Larson R., Leick M. B., Bailey S. R., Bouffard A. A., Millar D., Gallagher K., .(2024). Stealth transgenes enable CAR-T cells to evade host immune responses. J ImmunoTher Cancer. 12(5): e008417. 10.1136/jitc-2023-008417 PMC11086422 38724463

[16] Zhao Y., Chuai Y., Fu K., Han Y., Gao N., Nie G. and Chen Y. (2026). Synthetic biology integrated with material science paves the way for next-generation smart cell therapies. Molecular Therapy Oncology. 34(1): 201157 10.1016/j.omton .2026.201157 41809367 PMC12969017

[17] Zhou Y., Kong D., Wang X., Yu G., Wu X., Guan N., Weber W. and Ye H. (2021). A small and highly sensitive red/far-red optogenetic switch for applications in mammals. Nat Biotechnol. 40(2): 262 272 272. 10.1038/s41587-021-01036-w 34608325

[18] Park J., Andrade B., Seo Y., Kim M. J., Zimmerman S. C. and Kong H. (2018). Engineering the Surface of Therapeutic“Living” Cells. Chem Rev. 118(4): 1664 1690 1690. 10.1021/acs.chemrev .7b00157 29336552 PMC8243527

[19] Shi C., Chen T., Li Y., Li W., Shen Y., Cai K., Wang M. and Chen Y. (2025). Encapsulation of individual mammalian cells as a cell-based drug delivery carrier for lung cancer treatment. J Controlled Release. 378: 209 220 220. 10.1016/j.jconrel .2024.12.013 39662681

[20] Adebowale K., Liao R., Suja V. C., Kapate N., Lu A., Gao Y. and Mitragotri S. (2023). Materials for Cell Surface Engineering. Adv Mater. 36(43): e202210059. https://doi.org/10.1002/adma.202210059 36809574

[21] Yang Q., Zhao J., Muhammad A., Tian L., Liu Y., Chen L. and Yang P. (2022). Biopolymer coating for particle surface engineering and their biomedical applications. Mater Today Bio. 16: 100407 10.1016/j.mtbio .2022.100407 PMC9450159 36090610

[22] Shi P. and Wang Y. (2021). Synthetic DNA for cell-surface engineering. Angewandte Chemie International Edition, 60(21), 11580–11591. 10.1002/anie.202010278

[23] Zhou Y., Liu Y., Sun H. and Lu Y. (2025). Creating novel metabolic pathways by protein engineering for bioproduction. Trends Biotechnol. 43(5): 1094 1103 1103. 10.1016/j.tibtech .2024.10.017 39632163 PMC12064402

[24] Chen T. Y., Chang C. Y., Xu L., Wen T. C., Lin Y. H., Peng C. L., Yeh Y. Q., Chen C. H., Tsai I. L., Chuang K. H., .(2026). Short-chain dense brush PEGylation on rigid nanocarriers overcomes anti-PEG antibody recognition for immune-stealth drug delivery. Biomaterials. 328: 123854 10.1016/j.biomaterials .2025.123854 41265178

[25] Luo B., Wang S., Song X., Chen S., Qi Q., Chen W., Deng X., Ni Y., Chu C., Zhou G., .(2024). An Encapsulation‐Free and Hierarchical Porous Triboelectric Scaffold with Dynamic Hydrophilicity for Efficient Cartilage Regeneration. Adv Mater. 36(27): e202401009. https://doi.org/10.1002/adma.202401009 38548296

[26] Zhao Y., Li R., Han Y., Shi C., Lee K., Nie G. and Chen Y. (2026). On-demand cancer immunotherapy via single-cell encapsulation of synthetic circuit–engineered cells. Sci Adv. 12(3): eaea3573. https://doi.org/10.1126/sciadv.aea3573 PMC12802821 41533781

